# Gd-EOB MRI for HCC subtype differentiation in a western population according to the 5^th^ edition of the World Health Organization classification

**DOI:** 10.1007/s00330-023-09669-y

**Published:** 2023-04-28

**Authors:** Timo A. Auer, Sebastian Halskov, Uli Fehrenbach, Nora F. Nevermann, Uwe Pelzer, Raphael Mohr, Bernd Hamm, Wenzel Schöning, David Horst, Jana Ihlow, Dominik Geisel

**Affiliations:** 1 Department of Radiology, Charité - Universitätsmedizin Berlin, Augustenburger Platz 1, 13353 Berlin, Germany; 2 Berlin Institute of Health (BIH), Anna-Louisa-Karsch-Straße 2, 10178 Berlin, Germany; 3 Department of Surgery – CVK/CCM, Charité - Universitätsmedizin Berlin, Augustenburger Platz 1, 13353 Berlin, Germany; 4 Department of Hematology, Oncology and Cancer Immunology, Charité - Universitätsmedizin Berlin, Augustenburger Platz 1, 13353 Berlin, Germany; 5 Department of Hepatology and Gastroenterology, Charité - Universitätsmedizin Berlin, Augustenburger Platz 1, 13353 Berlin, Germany; 6 Institute of Pathology, Charité - Universitätsmedizin Berlin, corporate member of Freie Universität Berlin and Humboldt-Universität Zu Berlin, Charitéplatz 1, 10117 Berlin, Germany

**Keywords:** Liver, Magnetic resonance imaging, Hepatocellular carcinoma, Gadoxetic acid

## Abstract

**Objectives:**

To investigate the value of gadoxetic acid (Gd-EOB)–enhanced magnetic resonance imaging (MRI) for noninvasive subtype differentiation of HCCs according to the 5^th^ edition of the *WHO Classification of Digestive System Tumors* in a western population.

**Methods:**

This retrospective study included 262 resected lesions in 240 patients with preoperative Gd-EOB-enhanced MRI. Subtypes were assigned by two pathologists. Gd-EOB-enhanced MRI datasets were assessed by two radiologists for qualitative and quantitative imaging features, including imaging features defined in LI-RADS v2018 and area of hepatobiliary phase (HBP) iso- to hyperintensity.

**Results:**

The combination of non-rim arterial phase hyperenhancement with non-peripheral portal venous washout was more common in “not otherwise specified” (nos-ST) (88/168, 52%) than other subtypes, in particular macrotrabecular massive (mt-ST) (3/15, 20%), chromophobe (ch-ST) (1/8, 13%), and scirrhous subtypes (sc-ST) (2/9, 22%) (*p* = 0.035). Macrovascular invasion was associated with mt-ST (5/16, *p* = 0.033) and intralesional steatosis with steatohepatitic subtype (sh-ST) (28/32, *p* < 0.001). Predominant iso- to hyperintensity in the HBP was only present in nos-ST (16/174), sh-ST (3/33), and clear cell subtypes (cc-ST) (3/13) (*p* = 0.031). Associations were found for the following non-imaging parameters: age and sex, as patients with fibrolamellar subtype (fib-ST) were younger (median 44 years (19–66), *p* < 0.001) and female (4/5, *p* = 0.023); logarithm of alpha-fetoprotein (AFP) was elevated in the mt-ST (median 397 µg/l (74–5370), *p* < 0.001); type II diabetes mellitus was more frequent in the sh-ST (20/33, *p* = 0.027).

**Conclusions:**

Gd-EOB-MRI reproduces findings reported in the literature for extracellular contrast-enhanced MRI and CT and may be a valuable tool for noninvasive HCC subtype differentiation.

**Clinical relevance statement:**

Better characterization of the heterogeneous phenotypes of HCC according to the revised WHO classification potentially improves both diagnostic accuracy and the precision of therapeutic stratification for HCC.

**Key Points:**

*• Previously reported imaging features of common subtypes in CT and MRI enhanced with extracellular contrast agents are reproducible with Gd-EOB-enhanced MRI.*

*• While uncommon, predominant iso- to hyperintensity in the HBP was observed only in NOS, clear cell, and steatohepatitic subtypes.*

*• Gd-EOB-enhanced MRI offers imaging features that are of value for HCC subtype differentiation according to the 5* ^*th*^ *edition of the WHO Classification of Digestive System Tumors.*

**Supplementary Information:**

The online version contains supplementary material available at 10.1007/s00330-023-09669-y.

## Introduction

Hepatocellular carcinoma (HCC) is one of the leading causes of cancer-related mortality worldwide. Despite advances in all specialties and improved surveillance programs, approximately 65% of all HCCs are inoperable at diagnosis [1–3].

**Fig. 1:**
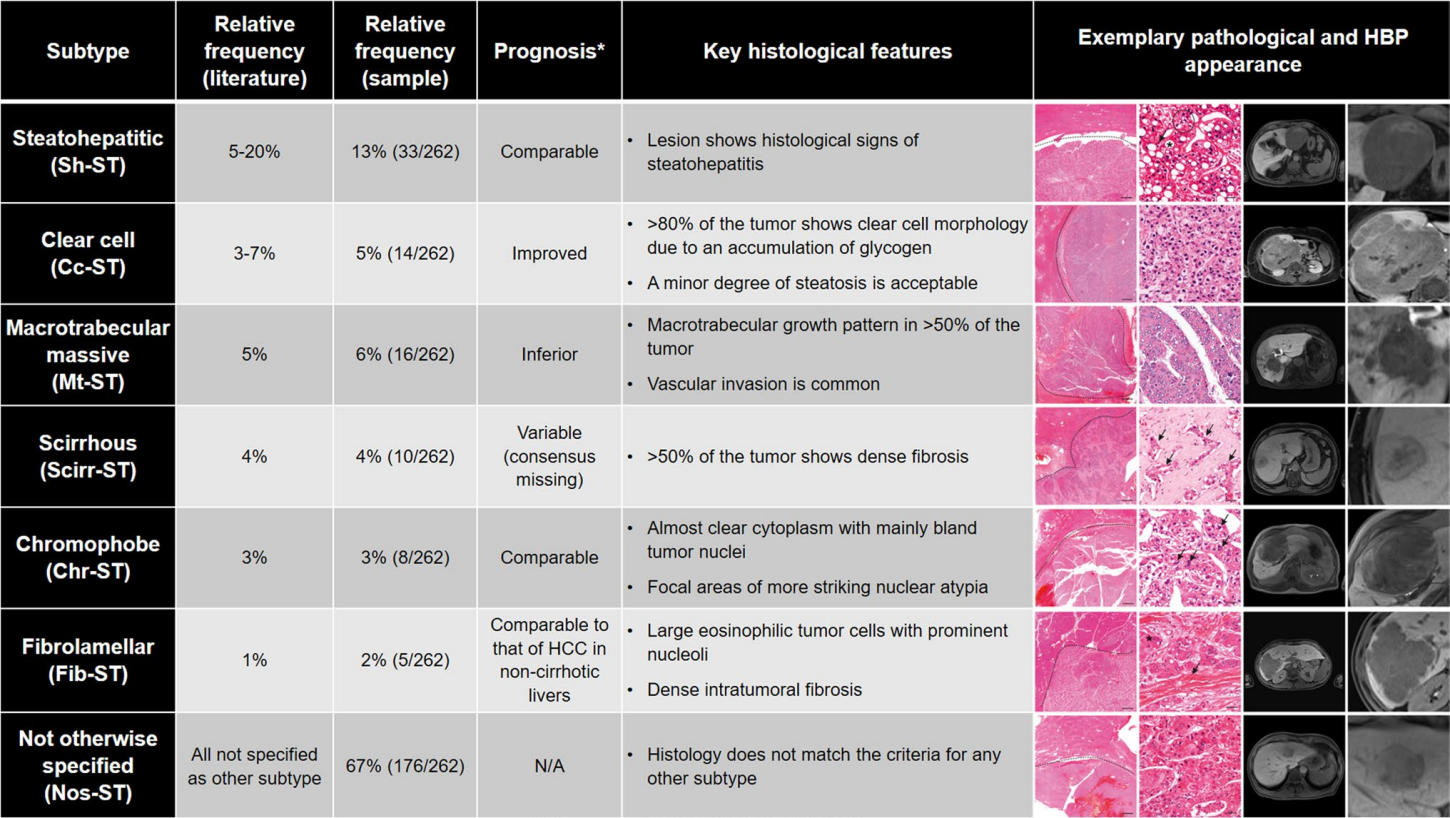
Overview of all HCC subtypes: relative frequency in the literature, frequency in our study population, related prognosis, key histological features, and HBP MRI appearance. *Compared to HCC of nos-ST

Published in 2019, the current *World Health Organization (WHO) Classification of Digestive System Tumors* distinguishes between eight instead of two histopathological subtypes [4, 5] (Fig. 1). This finer classification represents increasing knowledge of tumor biology and prognosis of subtypes [6–8]. This is particularly significant because histopathological properties, such as microvascular invasion, can predict prognosis and treatment response [9]. However, clear treatment recommendations according to histopathological subtypes have not been established. On the one hand, this may be due to a lack of data on progression-free and overall survival after surgical or systemic therapies from large-scale studies. On the other hand, broadly accepted noninvasive diagnostic features to identify the new subtypes are lacking.

In treatment algorithms of HCC, this poses a dilemma since HCC is the only tumor entity that may be confidently diagnosed as HCC in high-risk patients based on its imaging hallmarks [3, 4]. MRI with use of a hepatocyte-specific contrast agent such as gadobenate dimeglumine (MultiHance, Bracco Imaging) or gadoxetic acid (Gd-EOB; Primovist or Eovist, Bayer Pharma) is a cornerstone in the imaging of focal liver lesions [3]. Visualizing both vascularity and hepatocyte function, it provides an additional dimension of diagnostic information compared to MRI enhanced with extracellular contrast agents (ECAs) and has demonstrated utility for identifying macrotrabecular massive and steatohepatitic HCC [7, 10, 11]. There may exist further potential of Gd-EOB-enhanced MRI for identifying subtypes of HCC. For instance, predominant iso- to hyperintensity in the hepatobiliary phase (HBP) is a distinctive imaging feature that allows for precise subtyping of hepatocellular adenomas (HCAs) [12–18]. HBP hyperintensity has also been reported in 8.8–14% of HCCs [19–21]. The purpose of this study was therefore to investigate the value of Gd-EOB-enhanced MRI for HCC subtype differentiation in a western population according to the 5^th^ edition of the *WHO Classification of Digestive System Tumors*.

## Methods

### Design

This is an institutional review board–approved retrospective, non-confirmatory and explorative single-center study (internal registration number: EA1/323/20*)*. The requirement for informed consent was waived due to the retrospective nature of the study. The study protocol conforms to the ethical guidelines of the 2002 Declaration of Helsinki.

### Patients

Consecutive patients were retrospectively identified from a prospectively maintained surgical database and had to meet the following criteria for inclusion:

- Surgical resection of HCC between January 2010 and January 2022
- Preoperative Gd-EOB-enhanced MRI showing therapy-naïve HCC lesions
- Histopathological confirmation in accordance with the 5^th^ edition of the *WHO Classification of Digestive System Tumors*

Patients were excluded according to these criteria:

- Inadequate HBP sequences, where liver parenchyma is not unequivocally hyperintense to blood vessels
- No fat saturation available in HBP sequences
- Severe artifacts in HBP sequences

Lesions were excluded according to these criteria:

- Indeterminate subtype.
- A lesion of the same subtype was already included from the same patient, to avoid overrepresentation of potentially metastatic nodules. Larger lesions were preferred.

### Clinical parameters

Clinical parameters including age, gender, tumor recurrence, signs of elevated portal venous pressure, laboratory data, Eastern Cooperative Oncology Group performance status, Child-Pugh grade, and risk factors for HCC were recorded.

### Histopathology

**Fig. 2:**
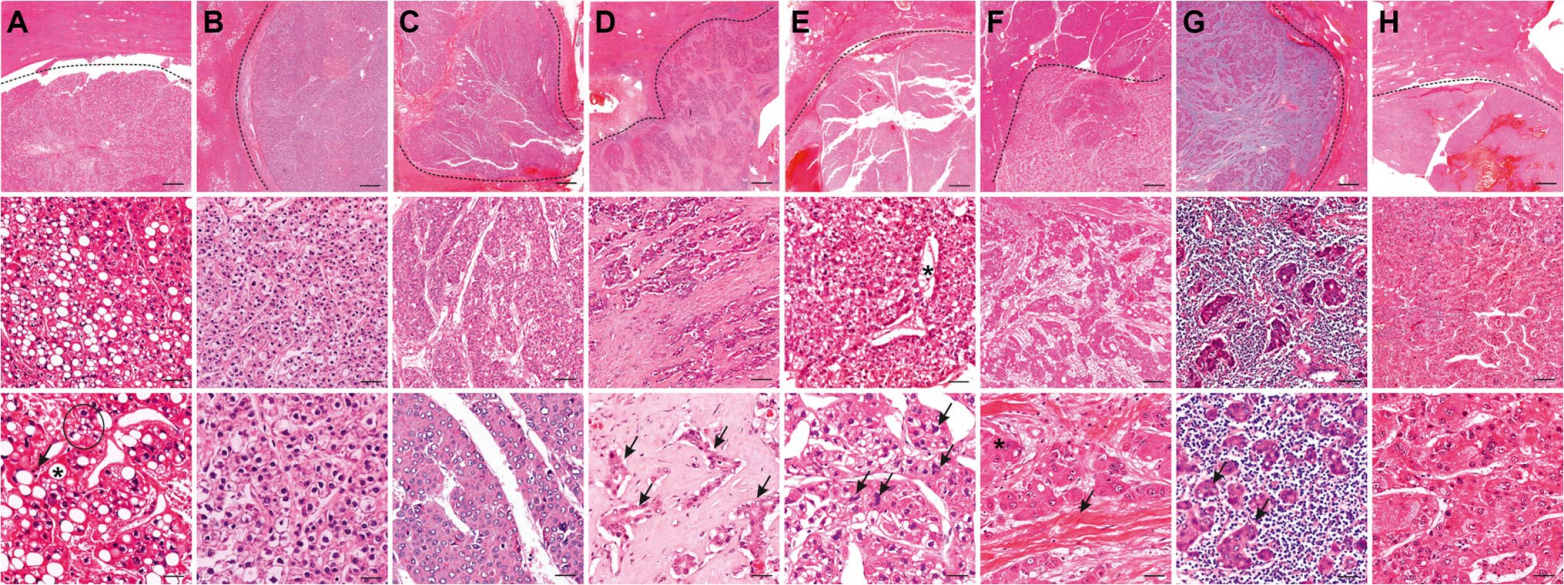
Hematoxylin and eosin stains of surgically resected HCC lesions included in this study. Each column represents one subtype of HCC, with magnification increasing from the top to the bottom rows. The dashed line marks the border between normal liver tissue and tumor. Scale bars: 500 µm, 100 µm, 50 µm. **A** *sh-ST*: Tumor cells contain fat vacuoles (*) and are ballooned (circle). There is marked pericellular fibrosis and chronic inflammation (arrow). **B** *cc-ST*: Tumor cells show abundant clear cytoplasm and bland round nuclei. Clear cell changes are present in at least 80% of the tumor. **C** *mt-ST*: This tumor grows predominantly (> 50%) in thick trabeculae that consist of sheets thicker than 6–10 tumor cells. In this case, most areas show high-grade nuclear atypia. **D** *sc-ST*: This tumor is characterized by abundant fibrous stroma consisting of thick fibrous septa that separate nests of poorly differentiated tumor cells. A fibrous tumor capsule is absent. Fibrosis is present in at least 50% of the tumor. **E** *chr-ST*: The tumor cells contain a smooth chromophobic, slightly eosinophilic cytoplasm and mostly small nuclei with low-grade changes and small inconspicuous nucleoli. Cyst-like spaces are found in between the tumor cells (*). However, in some areas, tumor cells show marked nuclear anaplasia (arrows). **F** *fib-ST*: The tumor has a trabecular appearance and consists of sheets or cords of large polygonal cells with abundant eosinophilic oncocytic cytoplasm, due to plenty of mitochondria (*). Nuclei show coarse chromatin and macronucleoli. In the interstitial space, there are dense collagen bundles arranged in parallel lamellae (arrow). **G** *Lymphocyte-rich HCC (excluded from analysis)*: This rare tumor consists of islets of pleomorphic tumor cells with atypical nuclei that are surrounded by a large number of lymphocytes. Lymphocytes outnumber and focally invade tumor cells. **H** *nos-ST*: The tumor predominantly shows a trabecular growth pattern with focal pseudoglandular changes and hemorrhages. The cytoplasm is deeply eosinophilic and has focal hyaline bodies; nuclear atypia is moderate. The normal liver tissue shows a regular structure

Two pathologists (J.I.,6 years of experience, and D.H., 18 years of experience) blinded to clinical and radiological findings independently reviewed all liver specimens to determine the subtype of each HCC using the criteria published in the 5^th^ edition of the WHO classification (citation Blue Book see Introduction) and by the Armed Forces Institute of Pathology [22]. For each sample, 3-µm FFPE slides stained for hematoxylin-eosin, periodic-acid Schiff reaction, Gomori-, Prussian blue, Fouchet, and chromotrope aniline blue stains were available from previous diagnostic procedures. In some cases (77/262), immunohistochemical stains for CK7 (Dako 1:1000, OV-TL 12/30), HepPar1 (1:100, Dako, OCH1E5), AFP (1:100, Epitomics, EP209), Glypican 3 (1:100, Zytomed, 1G12), Arginase 1 (1:500, Proteintech, McAB), glutamin synthetase (1:250, Merck Millipore, GS-6), polyclonal CEA (1:1000, BioGenex, TF3H8-1), EMA (1:100, Dako, E29), AFP (1:100, Epitomics, EP209), CD10 (1:5, Leica, 56C6), CD34 (1:50, Epitomics, EP88), or CD31 (1:25, Dako, JC/70A) were added for clarifying the diagnosis, subtyping, or assessment of vascular invasion. Immunohistochemical staining was performed using an automated Ventana BenchMark XT immunostainer (Ventana Medical Systems Inc.). Detailed criteria for histological subtyping are shown in Fig. 1 [4, 23, 24]. and exemplary lesions are shown in Fig. 2. Lesions with an indeterminate subtype were excluded from the analysis. Tumor grading was performed using the Edmondson/Steiner Classification [25]. Additionally, noncancerous liver tissue was evaluated for steatosis and cirrhosis. Severity of steatosis was graded as percentage of fat vacuoles per specimen surface (grade 0:  < 5%, grade 1: 5–33%, grade 2: 34–66%, grade 3:  > 66%) [26]. Cirrhosis was graded according to Desmet et al [27].

### Imaging

MRI was performed at 1.5 or 3.0 T using phased-array body coils. The standard imaging protocols included precontrast T2-weighted (T2w) sequences with and without fat saturation (FS), T1-weighted sequences (T1w) with and without FS (including in-/opposed-phase technique), and diffusion-weighted imaging (DWI) at *B* values of 50 and 800. After intravenous administration of Gd-EOB (0.025 mmol/kg body weight; manual injection at a flow rate of 1–2 mL/s, followed by a saline flush), multiphase T1w 3D sequences with FS were acquired during breath hold (arterial phase with a fixed delay of 15 s, portal venous phase with 50-s delay, and transitional phase with 90–120 s delay/transitional). 3D T1w FS imaging was repeated in the hepatobiliary phase 20 min after contrast administration. A detailed overview of our imaging parameters can be found in the electronic supplementary materials.

### Analysis

#### Reading

Subjective image analysis was performed by two board-certified radiologists with a core expertise in abdominal imaging and liver MRI (T.A.A.: 7 years of experience; D.G.: 13 years of experience). Images were read in consensus, with reliability analysis performed for subjective rating of intralesional Gd-EOB uptake area as the reliability of this imaging feature is not yet well studied. Both readers have previous experience in evaluating Gd-EOB uptake area.

#### Missing data

MRI sequences were excluded from subjective analysis if artifacts did not permit confident assessment of qualitative imaging features. Arterial phase T1w sequences were excluded from analysis if they were mistimed. Lesions with artifacts were excluded from quantitative assessment of enhancement in the respective phases.

#### Qualitative imaging parameters

**Fig. 3:**
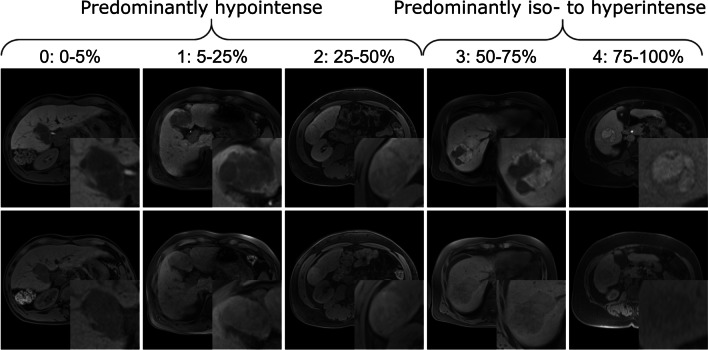
5-point scale for the subjective rating of Gd-EOB uptake by lesion area in the HBP. A lesion with a score of 3 or 4 is considered “predominantly iso- to hyperintense,” meaning that 50% or more of the total lesion area is iso- or hyperintense relative to the liver parenchyma in the HBP. Top row: HBP images; bottom row: precontrast T1-weighted images

All recorded imaging parameters are displayed in Table 1*.* Definitions are derived from CT/MRI LI-RADS v2018 [28]. Signal intensity in T1 and T2 sequences was subjectively rated relative to liver parenchyma according to the 5-point scale in Table 2. Intralesional Gd-EOB uptake in the HBP was rated as a percentage of intralesional iso- to hyperintensity in HBP on a 5-point scale (0, 0–5%; 1, 5–25%; 2, 25–50%; 3, 50–75%; and 4,  > 75%) (Fig. 3) [16]. Lesions with Gd-EOB uptake scores of 0–2 were classified as “Predominantly hypointense” and lesions with scores of 3–4 as “Predominantly iso- to hyperintense” similar to previous studies [16]. This is in line with the WHO classification, which subclassifies HCC according to the predominant histological component.

**Table 1:** Parameters assessed in qualitative image analysis

Qualitative imaging parameters	Possible values
Lesion size	Maximum diameter in mm in any plane
Arterial phase hyperenhancement (APHE)	Rim/non-rim/none
Portal venous phase “washout”	Peripheral/non-peripheral/none
Pseudocapsule in arterial phase	Yes/no
Nodule-in-nodule appearance	Yes/no
Macrovascular invasion	Yes/no
Diffusion restriction	Yes/no
Apparent diffusion coefficient (ADC)	Measured in ADC map
Intralesional steatosis	Yes/no
Intralesional hemorrhage	Yes/no
Mosaic architecture	T1w/T2w/none
Signal intensity in T1w, T2w, and dynamic phase sequences	*0 to 4 (see *Table 2*)*
Gd-EOB uptake area	*0 to 4 (see *Fig. 3*)*

**Table 2:** 5-point scale for the subjective rating of intralesional signal intensity in HCC

Intralesional signal intensity	Definition
0: Hypointense	Markedly lower intensity than liver parenchyma
1: Isointense to hypointense	Slightly lower intensity than liver parenchyma
2: Isointense	Same intensity as liver parenchyma
3: Isointense to hyperintense	Slightly higher intensity than liver parenchyma
4: Hyperintense	Markedly higher intensity than liver parenchyma

#### Quantitative imaging parameters

Two-dimensional circular regions of interest (ROIs) were placed by T.A.A. in an unenhanced T1-weighted fat-saturated sequence and in each dynamic phase sequence to quantify signal intensity and enhancement of lesions relative to liver parenchyma. Lesional ROIs were placed in enhancing areas. Parenchymal ROIs were placed near each lesion at a distance of approximately 1 to 2 cm from the lesion ROI in areas with homogeneous signal and no apparent blood vessels. Both ROIs were placed in the same position in each phase. Enhancement was defined as the difference in signal intensity compared with the unenhanced phase. The ratio between lesion and liver enhancement was calculated for each phase according to the formula shown below:

$$\frac{{Dynamic \;phase \;lesion \;signal \;intensity - Precontrast \;lesion \;signal \;intensity}}{{Dynamic \;phase \;liver \;signal \;intensity - Precontrast \;liver \;signal \;intensity}}$$

Apparent diffusion coefficients were determined by placing ROIs in homogenous intralesional areas within the respective maps.

### Statistics

All statistical analyses were performed using XLSTAT statistical and data analysis solution (Addinsoft) and IBM SPSS Statistics for Windows, version 28.0 (IBM Corp.). Descriptive statistics were carried out for all variables. Proportional distributions of categorical and ordinal variables among subtypes were compared with Fisher’s exact test or a Monte Carlo estimation, depending on the complexity of the calculations. Means of continuous variables were compared between groups using ANOVA if normal distribution and homoscedasticity were assumed. Nonparametric continuous variables were transformed logarithmically if this resulted in a normal distribution. Central tendencies of nonparametric continuous variables were compared between groups with the Kruskal-Wallis test. Inter-reader variability was tested by means of Cohen’s kappa test. A two-sided *p* value of less than 0.05 was considered statistically significant.

## Results

### Selection

**Fig. 4:**
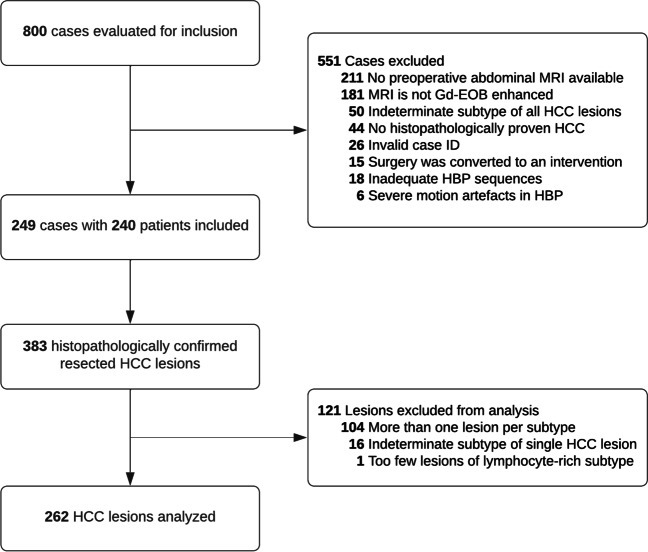
Flow chart detailing the results of the selection procedure

Out of 800 cases initially evaluated for inclusion, 249 cases were included in this study. Eighteen cases were excluded due to inadequate HBP sequences. A total of 383 histopathologically confirmed, surgically resected HCC lesions were identified. Finally, 262 lesions in 240 patients were included in analysis after excluding 16 lesions with an indeterminate subtype, 104 lesions in cases where more than one lesion per subtype was present in the liver, and a single lesion of the lymphocyte-rich subtype because of its statistical irrelevance. Figure 4 provides an overview of the selection procedure. The median interval between preoperative MRI and resection was 34 days (12–58).

### Histopathological analysis

Subtype frequencies are displayed in Fig. 1. Intra-patient heterogeneity with multiple subtypes within the same liver was found in 32/240 patients (13.3%). Intra-lesion heterogeneity with regions representing 2 or more subtypes excluding nos-ST was found in 20/262 lesions (7.6%). Inter-reader variability between pathologists was excellent at Cohen’s kappa 0.951 (0.916–0.986).

### Clinical data

The median size of all lesions was 33 mm (20–55) and ranged from 31 mm (17–47) in the mt-ST subgroup to 50 mm (26–108) in the fib-ST subgroup (*p* = 0.071). The median age of the total study population was 66 years (59–72) with a significant difference between subtype groups (*p* < 0.001) as patients with a fib-ST were significantly younger with a median age of 44 years (19–66). While 72% (188/262) of lesions occurred in male patients, a significantly higher proportion with 80% (4/5) of fib-ST was found in women. Furthermore, the mt-ST (female: 44% (7/16)) and the chr-ST (female: 50% (4/8)) affected nearly equal numbers of women as men (*p* = 0.002) (Table 3). Cirrhosis and signs of portal hypertension were present in 158/258 (61%) and 65/262 (25%) of all patients, respectively. Apart from the fib-ST, which occurred in patients without any liver pathologies or known risk factors (*p* < 0.001), and the sh-ST, which occurred significantly more frequently in patients with diabetes mellitus type II (*p* = 0.027), no other significant differences in the grade or etiology of underlying liver cirrhosis were found between HCC subtypes. AFP (median of the whole cohort: 8 µg/l (4–82)) was the only laboratory parameter showing a significant difference after logarithmic transformation with a median of 397 µg/l (74–5370) for the mt-ST versus 208 µg/l (5–3167) for the chr-ST (*p* < 0.001). The results are compiled in Table 3.

**Table 3:** Patient characteristics and ris factors by HCC lesion subtype in the study population

Patient characteristics	All (*n* = 262)	NOS (*n* = 176)	Fibrolamellar (*n* = 5)	Macrotrabecular massive (*n* = 16)	Clear cell (*n* = 14)	Steatohepatitic (*n* = 33)	Scirrhous (*n* = 10)	Chromophobe (*n* = 8)	*p* value
Age (y) median (IQR)	66 (59–72)	66 (59–73)	44 (19–66)	63 (54–71)	68 (57–78)	65 (54–72)	64 (58–71)	71 (67–74)	< 0.001*
Sex	M: 72% (188/262) F: 28% (74/262)	M: 74% (130/176) F: 26% (46/176)	M: 20% (1/5) F: 80% (4/5)	M: 56% (9/16) F: 44% (7/16)	M: 57% (8/14) F: 43% (6/14)	M: 85% (28/33) F: 15% (5/33)	M: 80% (8/10) F: 20% (2/10)	M: 50% (4/8) F: 50% (4/8)	0.023*
Recurrence	16% (43/262)	17% (30/176)	20% (1/5)	13% (2/16)	21% (3/14)	18% (6/33)	10% (1/10)	0% (0/8)	0.913
Liver transplantation	23% (59/262)	23% (41/176)	0% (0/5)	13% (2/16)	7% (1/14)	36% (12/33)	20% (2/10)	13% (1/8)	0.286
Increased PV pressure	25% (65/262)	26% (46/176)	0% (0/5)	13% (2/16)	29% (4/14)	24% (8/33)	20% (2/10)	38% (3/8)	0.741
Ascites	7% (19/261)	7% (12/176)	0% (0/5)	13% (2/16)	7% (1/14)	6% (2/33)	10% (1/10)	14% (1/7)	0.772
AFP (µg/l) median (IQR)	8 (4–82)	8 (5–81)	30 (9–15,846)	397 (74–5370)	5 (3–6)	6 (3–11)	5 (3–24)	208 (5–3167)	< 0.001*
ECOG performance status	0: 50% (50/101) 1: 45% (45/101) 2 + : 6% (6/101)	0: 55% (37/67) 1: 42% (28/67) 2 + : 3% (2/67)	0: 50% (1/2) 1: 50% (1/2) 2 + : 0% (0/2)	0: 50% (3/6) 1: 33% (2/6) 2 + : 17% (1/6)	0: 25% (2/8) 1: 63% (5/8) 2 + : 13% (1/8)	0: 30% (3/10) 1: 50% (5/10) 2 + : 20% (2/10)	0: 50% (2/4) 1: 50% (2/4) 2 + : 0% (0/4)	0: 50% (2/4) 1: 50% (2/4) 2 + : 0% (0/4)	0.413
Child-Pugh grade (from report)	A: 81% (78/96) B: 11% (11/96) C: 7% (7/96)	A: 82% (58/71) B: 13% (9/71) C: 6% (4/71)	–	A: 86% (6/7) B: 0% (0/7) C: 14% (1/7)	A: 75% (3/4) B: 0% (0/4) C: 25% (1/4)	A: 78% (7/9) B: 22% (2/9) C: 0% (0/9)	A: 75% (3/4) B: 0% (0/4) C: 25% (1/4)	A: 100% (1/1) B: 0% (0/1) C: 0% (0/1)	0.451
Liver pathology	N: 5% (13/258) S: 9% (22/258) F: 25% (65/258) C: 61% (158/258)	N: 3% (5/174) S: 9% (15/174) F: 23% (40/174) C: 66% (114/174)	N: 100% (5/5) S: 0% (0/5) F: 0% (0/5) C: 0% (0/5)	N: 6% (1/16) S: 6% (1/16) F: 38% (6/16) C: 50% (8/16)	N: 17% (2/12) S: 8% (1/12) F: 33% (4/12) C: 42% (5/12)	N: 0% (0/33) S: 9% (3/33) F: 24% (8/33) C: 67% (22/33)	N: 0% (0/10) S: 10% (1/10) F: 30% (3/10) C: 60% (6/10)	N: 0% (0/8) S: 13% (1/8) F: 50% (4/8) C: 38% (3/8)	0.001*
Risk factors									
Diabetes type 2	37% (98/262)	35% (62/176)	0% (0/5)	19% (3/16)	43% (6/14)	61% (20/33)	30% (3/10)	50% (4/8)	0.027*
Alcoholism	31% (80/262)	30% (53/176)	0% (0/5)	44% (7/16)	21% (3/14)	30% (10/33)	30% (3/10)	50% (4/8)	0.517
Chronic hepatitis B	17% (44/262)	18% (31/176)	0% (0/5)	19% (3/16)	7% (1/14)	15% (5/33)	40% (4/10)	0% (0/8)	0.389
Chronic hepatitis C	24% (63/262)	27% (47/176)	0% (0/5)	31% (5/16)	7% (1/14)	21% (7/33)	10% (1/10)	25% (2/8)	0.493
Hepatocellular adenoma	3% (7/262)	2% (4/176)	0% (0/5)	6% (1/16)	7% (1/14)	3% (1/33)	0% (0/10)	0% (0/8)	0.522
Primary biliary cirrhosis	1% (5/262)	1% (4/176)	0% (0/5)	0% (0/16)	0% (0/14)	0% (0/33)	0% (0/10)	6% (1/8)	0.444
Other	4% (11/262)	5% (9/176)	0% (0/5)	0% (0/16)	0% (0/14)	3% (1/33)	10% (1/10)	0% (0/8)	0.822
None	16% (42/262)	15% (26/176)	100% (5/5)	13% (2/16)	21% (3/14)	9% (3/33)	20% (2/10)	13% (1/8)	0.002*

*IQR* interquartile range, *PV* portal venous, *ECOG* Eastern Cooperative Oncology Group, *AFP* alpha-fetoprotein; Pathology: *N* none, *S* steatosis, *F* fibrosis, *C* cirrhosis

### Image analysis

**Fig. 5:**
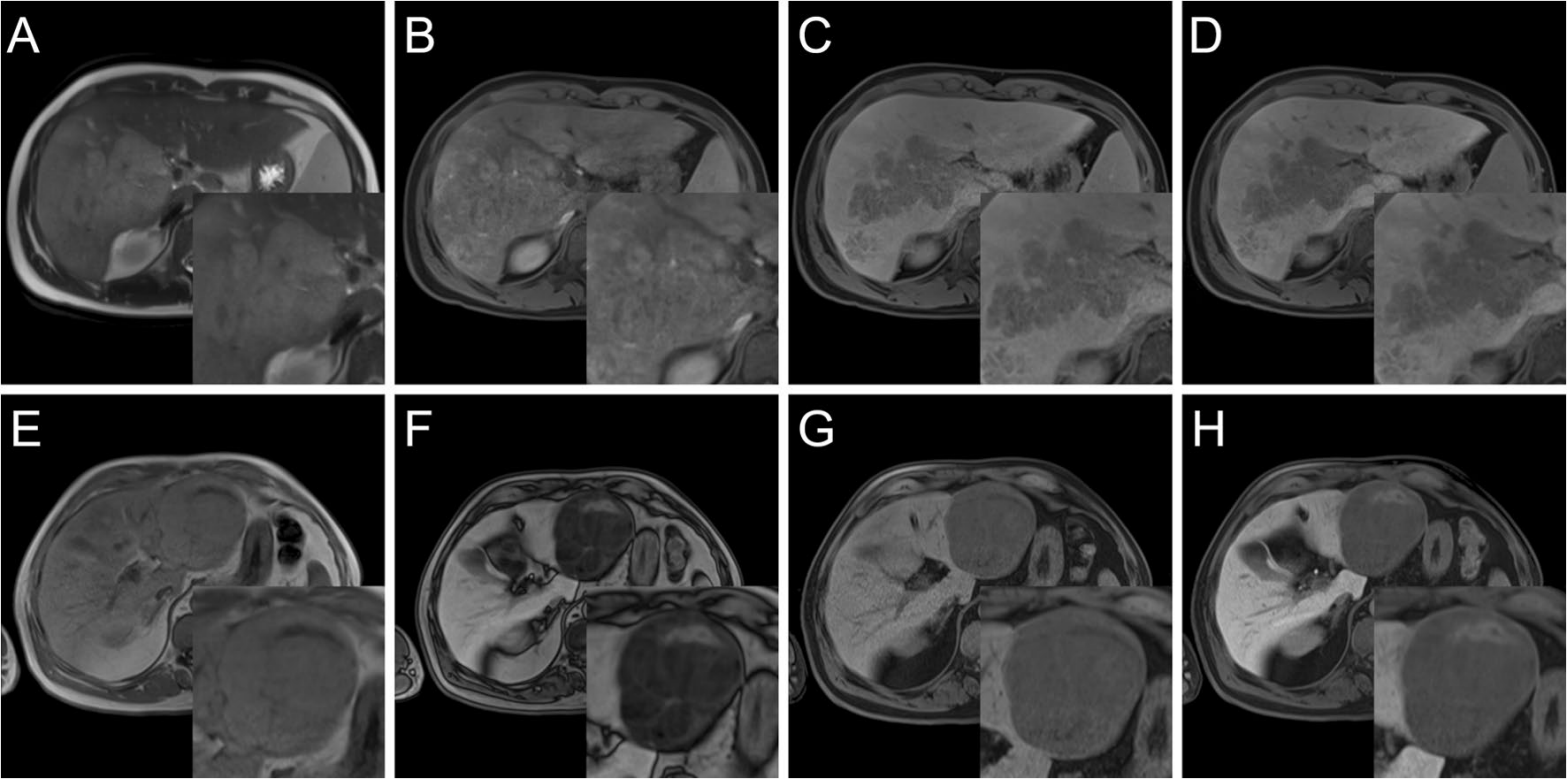
*Top row*: 21-year-old man with liver fibrosis and chronic hepatitis B infection. MRI shows a large HCC lesion of the mt-ST infiltrating the right liver lobe, with (**A**) marked hyperintensity of the lesion in T2-weighted image; (**B**) APHE in a heterogenous pattern, (**C**) “washout” and hypointense signal in the central portal vein during the venous phase indicating macrovascular invasion, and (**D**) marked Gd-EOB uptake deficiency intralesionally and to a lesser extent in much of the liver with the exception of segment I, further supporting macrovascular invasion. The histological images of this patient are presented in Fig. 2 column C. *Bottom row*: 71-year-old man with liver fibrosis and diabetes type II. Large HCC lesion of the sh-ST in the left liver lobe with a nearly ubiquitous drop in signal intensity between (**E**) in-phase and (**F**) opposed-phase images, indicating diffuse steatosis. Furthermore, compared to liver parenchyma and (**G**) the unenhanced T1-weighted images, (**H**) most of the lesion area becomes hypointense in the HBP. The histological images of this patient are presented in Fig. 2 column A

**Fig. 6:**
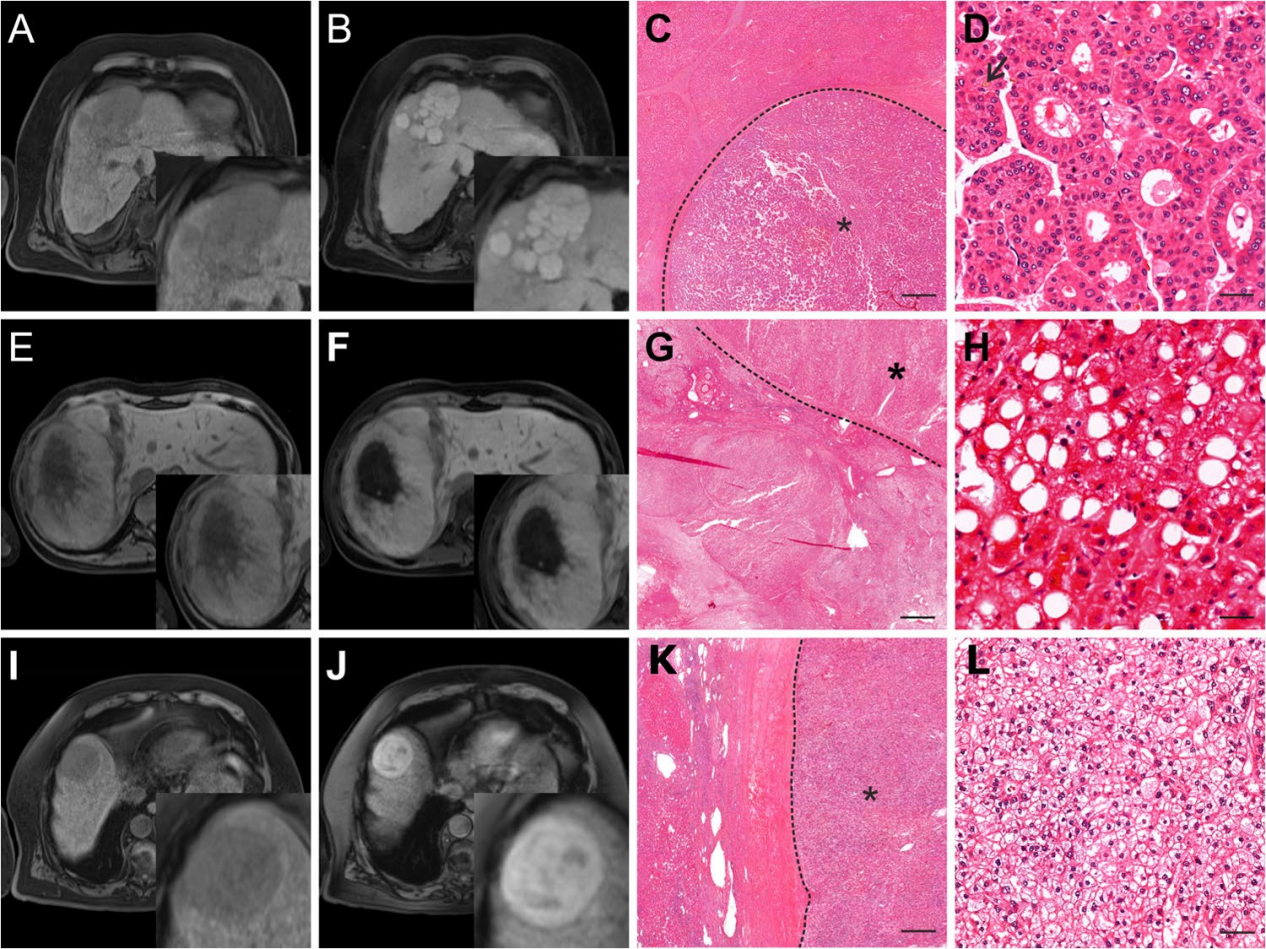
Three lesions showing iso- to hyperintensity in the HBP. Left column: precontrast T1-weighted sequence, middle left column: HBP, middle right and right columns: histopathology at low and high magnification (scale bars 500 µm and 50 µm, respectively). *Top row*: 69-year-old man with liver cirrhosis, diabetes type II, and a history of alcoholism. **A**, **B** Polycyclic HCC lesion of nos-ST in segment IV showing hyperintense nodules between hypointense septae. **C**, **D** * marks the tumor area. The tumor shows predominantly a pseudoglandular growth pattern that might be a correlate for hyperintensity. Tumor cells show mild nuclear atypia. Focally, bile production is visible (arrow). *Middle row*: 23-year-old woman with liver steatosis and no known risk factors for HCC. **E**, **F** Monstrous, well-differentiated HCC lesion of sh-ST throughout the right liver lobe, isointense to parenchyma in HBP and with a large central area of necrosis. **G**, **H** * marks the tumor area. This borderline neoplasia shows predominantly mild nuclear atypia and is mostly encapsulated. Tumor cells contain fat vacuoles and are ballooned. Also, few intratumoral lymphocytes are present. However, there are invasive areas with destroyed reticulin fibers and moderate cellular and nuclear atypia, which promoted diagnosis of HCC. In the surrounding liver tissue, severe steatosis is present. *Bottom row*: 82-year-old man with liver cirrhosis and diabetes type II. **I**, **J** Solitary HCC of cc-ST in segment VIII, hyperintense in HBP in a mosaic pattern and with a hypointense capsule. **K**, **L** * marks the tumor area. Tumor cells show clear cytoplasm and bland nuclei

The results of image analysis according to Tables 1 and 2 are compiled in Table 4. The typical enhancement pattern, consisting of non-rim APHE and non-peripheral portal venous washout, was significantly more common in nos-ST (88/168, 52%) compared to other subtypes, in particular ch-ST (1/8, 13%), sc-ST (2/9, 22%), and mt-ST (3/15, 20%) (*p* = 0.035). Intralesional steatosis was very common in sh-ST (88%, 28/32) and slightly more common in cc-ST (31%, 4/13) and mt-ST (27%, 4/15) compared to nos-ST (18%, 30/169) (*p* < 0.001). Macrovascular invasion was associated with the mt-ST, present in 5/16 (31%) compared to 12/176 (7%) of nos-ST (*p* = 0.031). Representative lesions of sh-ST and mt-ST exhibiting these features are shown in Fig. 5. Significant HBP enhancement was present in 8.5% (22/258) of lesions and only in the following subtypes: nos-ST: 9.2% (16/174), sh-ST: 9.1% (3/33), and cc-ST: 23.1% (3/13) (*p* = 0.031 in post hoc analysis) (Table 4 and Fig. 6). Among these predominantly iso- to hyperintense lesions, 6/22 (27.3%) were histologically graded as G1, 14/22 (63.6%) as G2, and 2/22 (9.1%) as G3. Transformation from HCA is known to have occurred in 2/22 of these lesions (11.1%), compared to 4/240 (1.7%) of all other lesions. There was a significant association between the enhancement ratio in HBP and visual classes of Gd-EOB uptake area due to a high enhancement ratio in class 4 (*p* = 0.001). Inter-reader agreement between the two radiologists for subjective rating of Gd-EOB uptake was good with a Cohen’s kappa value of 0.761 (0.694–0.828). The other parameters showed no significant differences (*p* > 0.05).

**Table 4:** Basic lesion characteristics by HCC lesion subtype in the study population. Signal intensity was subjectively rated according to the scale set out in Table 2. Enhancement ratio is the ratio between lesion and liver enhancement relative to the precontrast T1-weighted phase

Lesion characteristics	All (*n* = 262)	NOS (*n* = 176)	Fibrolamellar (*n* = 5)	Macrotrabecular massive (*n* = 16)	Clear cell (*n* = 14)	Steatohepatitic (*n* = 33)	Scirrhous (*n* = 10)	Chromophobe (*n* = 8)	*p* value
Size (mm) median (IQR)	33 (20–55)	32 (19–51)	50 (26–108)	31 (17–47)	49 (33–79)	32 (21–53)	47 (24–61)	44 (31–106)	0.071
Differentiation (Edm. St.)	1: 14% (35/256) 2: 68% (174/256) 3: 18% (47/256)	1: 14% (25/173) 2: 67% (116/173) 3: 18% (32/173)	1: 0% (0/4) 2: 50% (2/4) 3: 50% (2/4)	1: 0% (0/16) 2: 63% (10/16) 3: 38% (6/16)	1: 25% (3/12) 2: 67% (8/12) 3: 8% (1/12)	1: 21% (7/33) 2: 70% (23/33) 3: 9% (3/33)	1: 0% (0/10) 2: 80% (8/10) 3: 20% (2/10)	1: 0% (0/8) 2: 88% (7/8) 3: 13% (1/8)	0.202
Nodule-in-nodule appearance	18% (48/262)	18% (32/176)	20% (1/5)	25% (4/16)	14% (2/14)	15% (5/33)	10% (1/10)	38% (3/8)	0.749
Macrovascular invasion	8% (21/262)	7% (12/176)	20% (1/5)	31% (5/16)	7% (1/14)	3% (1/33)	0% (0/10)	13% (1/8)	0.033*
Intralesional hemorrhage	23% (59/262)	22% (38/176)	20% (1/5)	19% (3/16)	29% (4/14)	24% (8/33)	20% (2/10)	38% (3/8)	0.925
Intralesional steatosis	27% (69/251)	18% (30/169)	20% (1/5)	27% (4/15)	31% (4/13)	88% (28/32)	11% (1/9)	13% (1/8)	< 0.001*
Diffusion restriction	71% (130/182)	71% (92/130)	100% (2/2)	80% (8/10)	67% (6/9)	67% (14/21)	100% (4/4)	67% (4/6)	0.277
ADC median (IQR)	986 (867–1104)	954 (865–1084)	656 –	963 (748–1213)	1038 (987–1148)	998 (786–1140)	1028 (815–1106)	1063 (983–1247)	0.716
Mosaic architecture	T1: 14% (36/255) T2: 32% (82/255)	T1: 13% (22/171) T2: 29% (50/171)	T1: 0% (0/5) T2: 40% (2/5)	T1: 6% (1/16) T2: 31% (5/16)	T1: 29% (4/14) T2: 50% (7/14)	T1: 24% (8/33) T2: 33%(11/33)	T1: 0% (0/9) T2: 44% (4/9)	T1: 14% (1/7) T2: 43% (3/7)	0.198
Pseudocapsule	33% (78/240)	31% (51/163)	25% (1/4)	64% (9/14)	21% (3/14)	25% (7/28)	22% (2/9)	63% (5/8)	0.069
Arterial phase hyperenhancement (R: rim, N: non-rim)	R: 15% (37/248) N: 53% (132/248)	R: 13% (22/168) N: 59% (99/168)	R: 25% (1/4) N: 50% (2/4)	R: 33% (5/15) N: 27% (4/15)	R: 7% (1/14) N: 43% (6/14)	R: 10% (3/30) N: 57% (17/30)	R: 44% (4/9) N: 33% (3/9)	R: 13% (1/8) N: 13% (1/8)	0.087
PVP washout (P: peripheral, N: non-peripheral)	P: 2% (4/257) N: 70% (179/257)	P: 1% (2/174) N: 72% (125/174)	P: 0% (0/5) N: 60% (3/5)	P: 0% (0/16) N: 81% (13/16)	P: 0% (0/14) N: 57% (8/14)	P: 7% (2/30) N: 70% (21/30)	P: 0% (0/10) N: 60% (6/10)	P: 0% (0/8) N: 38% (3/8)	0.234
Typical enhancement pattern*	46% (114/247)	52% (88/168)	50% (2/4)	20% (3/15)	36% (5/14)	45% (13/29)	22% (2/9)	13% (1/8)	0.035
T1 signal intensity	0: 29% (77/262) 1: 34% (88/262) 2: 24% (64/262) 3: 10% (26/262) 4: 3% (7/262)	0: 27% (48/176) 1: 33% (58/176) 2: 25% (44/176) 3: 13% (22/176) 4: 2% (4/176)	0: 60% (3/5) 1: 40% (2/5) 2: 0% (0/5) 3: 0% (0/5) 4: 0% (0/5)	0: 56% (9/16) 1: 38% (6/16) 2: 6% (1/16) 3: 0% (0/16) 4: 0% (0/16)	0: 29% (4/14) 1: 43% (6/14) 2: 29% (4/14) 3: 0% (0/14) 4: 0% (0/14)	0: 21% (7/33) 1: 27% (9/33) 2: 36% (12/33) 3: 9% (3/33) 4: 6% (2/33)	0: 30% (3/10) 1: 30% (3/10) 2: 20% (2/10) 3: 10% (1/10) 4: 10% (1/10)	0: 38% (3/8) 1: 50% (4/8) 2: 13% (1/8) 3: 0% (0/8) 4: 0% (0/8)	0.424
T2 signal intensity	0: 0% (1/262) 1: 5% (13/262) 2: 20% (53/262) 3: 55% (143/262) 4: 20% (52/262)	0: 1% (1/176) 1: 5% (8/176) 2: 22% (39/176) 3: 56% (99/176) 4: 16% (29/176)	0: 0% (0/5) 1: 0% (0/5) 2: 0% (0/5) 3: 80% (4/5) 4: 20% (1/5)	0: 0% (0/16) 1: 0% (0/16) 2: 6% (1/16) 3: 44% (7/16) 4: 50% (8/16)	0: 0% (0/14) 1: 7% (1/14) 2: 14% (2/14) 3: 57% (8/14) 4: 21% (3/14)	0: 0% (0/33) 1: 6% (2/33) 2: 24% (8/33) 3: 45% (15/33) 4: 24% (8/33)	0: 0% (0/10) 1: 10% (1/10) 2: 20% (2/10) 3: 60% (6/10) 4: 10% (1/10)	0: 0% (0/8) 1: 13% (1/8) 2: 13% (1/8) 3: 50% (4/8) 4: 25% (2/8)	0.657
Gd-EOB uptake area	0: 30% (78/258) 1: 42% (109/258) 2: 19% (48/258) 3: 5% (13/258) 4: 4% (10/258)	0: 31% (54/174) 1: 43% (75/174) 2: 16% (28/174) 3: 5% (9/174) 4: 5% (8/174)	0: 60% (3/5) 1: 40% (2/5) 2: 0% (0/5) 3: 0% (0/5) 4: 0% (0/5)	0: 44% (7/16) 1: 44% (7/16) 2: 13% (2/16) 3: 0% (0/16) 4: 0% (0/16)	0: 23% (3/13) 1: 38% (5/13) 2: 15% (2/13) 3: 15% (2/13) 4: 8% (1/13)	0: 21% (7/33) 1: 42% (14/33) 2: 27% (9/33) 3: 6% (2/33) 4: 3% (1/33)	0: 20% (2/10) 1: 40% (4/10) 2: 40% (4/10) 3: 0% (0/10) 4: 0% (0/10)	0: 29% (2/7) 1: 29% (2/7) 2: 43% (3/7) 3: 0% (0/7) 4: 0% (0/7)	0.731
Enhancement ratio, arterial phase, mean (SD)	6.23 (9.72) (*n* = 181)	5.94 (8.21) (*n* = 116)	17.61 (22.51) (*n* = 3)	7.14 (6.67) (*n* = 15)	4.71 (6.46) (*n* = 12)	4.09 (4.72) (*n* = 22)	18.15 (30.97) (*n* = 7)	2.34 (0.92) (*n* = 7)	0.072
Enhancement ratio, portal venous phase, mean (SD)	1.41 (0.99) (*n* = 238)	1.39 (1.05) (*n* = 159)	2.11 (1.04) (*n* = 4)	1.62 (0.63) (*n* = 16)	1.18 (0.39) (*n* = 14)	1.28 (0.65) (*n* = 28)	2.18 (1.78) (*n* = 9)	1.21 (0.48) (*n* = 8)	0.207
Enhancement ratio, venous phase, mean (SD)	1.16 (0.8) (*n* = 233)	1.17 (0.87) (*n* = 155)	1.23 (0.19) (*n* = 5)	1.30 (0.44) (*n* = 16)	0.92 (0.45) (*n* = 14)	1.08 (0.74) (*n* = 27)	1.52 (1.07) (*n* = 8)	1.09 (0.5) (*n* = 8)	0.266
Enhancement ratio, HBP, mean (SD)	0.70 (0.54) (*n* = 226)	0.68 (0.53) (*n* = 153)	0.44 (0.16) (*n* = 5)	0.80 (0.79) (*n* = 15)	0.87 (0.81) (*n* = 10)	0.77 (0.47) (*n* = 28)	0.68 (0.31) (*n* = 8)	0.80 (0.15) (*n* = 7)	0.501

*IQR* interquartile range, *Edm. St.* Edmondson Steiner, *PVP* portal venous phase, *ADC* apparent diffusion coefficient, *SD* standard deviation, *HBP* hepatobiliary phase

^*^The typical enhancement pattern represents the combination of non-rim arterial phase hyperenhancement and non-peripheral portal venous phase washout

## Discussion

In summary, we were able to reproduce previously reported imaging features in ECA-enhanced MRI associated with common subtypes of HCC, including intralesional steatosis in sh-ST and macrovascular invasion in mt-ST, emphasizing their validity. Predominant iso- to hyperintensity in HBP was present only in a subset of HCC subtypes, to our knowledge the first time this imaging feature is reported in the context of the new WHO 5^th^ edition subtypes.

A key finding was that the combination of non-rim APHE and non-peripheral portal venous phase washout, a hallmark in imaging of HCC, was most prevalent in the nos-ST (*p* = 0.035). This may be an expected finding given differences in the histopathological architecture among subtypes, such as presence of fibrous stroma in sc-ST. Notably, Cannella et al [7] and Mulé et al [11] reported no significant differences among subtypes in this regard. We believe this discrepancy could be explained by the use of Gd-EOB. The ch-ST also stands out as only a single lesion showed typical enhancement, which is at odds with the scarce literature available on imaging features of this subtype and merits further investigation [29].

We found the mt-ST to be associated with macrovascular invasion (*p* = 0.033) and elevated AFP (*p* < 0.001). This is in line with the literature, where, for instance, Cannella et al reported these features as predictors for mt-ST in a study of 295 patients who underwent CE-CT and/or MRI enhanced with ECA or Gd-EOB [7, 30]. In other surgical cohorts, the mt-ST has been associated with a large tumor size upon diagnosis, with a commonly reported cut-off of 5 cm [7, 10, 11, 31, 32]. We were not able to reproduce this finding, however, as the median mt-ST size was 3.1 cm in our patients, with no significant difference between subtypes. We believe this could reflect regional and institutional variations in the management of HCC, including surveillance strategies and criteria for resection or liver transplantation [3]. It is a key finding that we were able to reproduce these features in a cohort of smaller, less advanced lesions.

The subset of 33 sh-ST HCCs in our study is a large sample of this subtype, especially under consideration of its western origin. We found the classical feature of intralesional steatosis in MRI to be strongly associated with the sh-ST (*p* < 0.001), consistent with the results of Inui et al, who observed intralesional fat in 80% of 20 sh-ST HCCs [33]. An important differential diagnosis may include cc-ST, which can undergo a metabolic shift from a glycogen-rich to a steatotic phenotype with cytoplasmatic fat [34]. Accordingly, we observed intralesional steatosis more commonly in cc-ST (31%) than in nos-ST (17%), but far less commonly than in sh-ST (88%).

Another subtype that stands out is the fib-ST. In our study, the fib-ST occurred more frequently in younger patients, but with a large interquartile range of 19–66 years (*p* < 0.001), in females (80%) (*p* = 0.023) and in the absence of known risk factors for HCC (*p* = 0.002). This is mostly in line with the literature, which describes two age-specific incidence peaks and a lower male-to-female ratio [35]. No specific imaging features were found for this subtype, however.

A subset of HCCs (8.5%) in our cohort were predominantly iso- to hyperintense in the HBP. The pathophysiological mechanism may be the initial overexpression of membrane transporters OATPB1/B3, which then gradually lose their function with progressive tumor dedifferentiation, giving HCCs their typical hypointense appearance. This is corroborated by Kitao et al [21] and Haimerl et al [36], who suggested that Gd-EOB uptake correlates with differentiation according to Edmondson and Steiner. High enhancement of nodules in HBP has been reported by Aoki et al [37] to be of value for predicting poor response to anti-PD-1/PD-L1 monotherapy for unresectable HCC, highlighting the potential of Gd-EOB to reflect immunological subclasses. Further groups have leveraged this imaging feature for precise subtyping of HCA, as the beta-catenin subtype shows preserved OATPB1/B3 expression and HBP hyperintensity [12–18].

All predominantly iso- to hyperintense HCCs in our collective were of the nos-ST (72.7%), sh-ST (13.6%), and cc-ST (13.6%). Both sh-ST and cc-ST are regarded as having a better prognosis than nos-ST [5]. This is in line with the findings of Kim et al [20], who reported that HCCs with higher HBP signal intensity had lower rates of microvascular invasion and more commonly showed peliotic changes. Kim et al concluded from their findings that patients with HCC with higher HBP signal intensity may have a more favorable outcome [20]. Gd-EOB-enhanced MRI may thus potentially be of added value not only for predicting treatment response and well-differentiated HCC, but also for identifying sh-ST, cc-ST, or nos-ST. However, we observed that the majority (72.7%) of lesions with high Gd-EOB uptake in our cohort were of intermediate or poor differentiation, potentially reflecting intra-tumor heterogeneity. Furthermore, this imaging feature was uncommon, indicating a need for further predictors. More studies investigating imaging appearance, histopathological features, and membrane transporter profiles are needed, including association with long-term outcomes.

A strength of our study is that we investigated a large western sample, thus directly addressing an important concern regarding Gd-EOB, namely that most data currently available were obtained in Eastern countries, where most HCCs arise in patients with a history of a hepatitis B virus infection and preserved liver function.

Some aspects of our method have to be critically discussed. Our cohort was recruited from a surgical database and therefore represents a subset of the high-risk population with earlier stages of HCC [3]. The pathological-radiological changes that occur during progressive dedifferentiation of HCC are well documented. Nakachi et al [38] showed that well- and poorly differentiated HCCs are likelier to have hypovascular regions on imaging. However, our center also offered surgery to patients with intermediate and advanced HCC in one quarter of cases, based on a size over 5 cm or not fulfilling Milan criteria. We believe this partially alleviated the selection bias inherent to a surgical cohort, albeit introducing heterogeneity.

Our study has further limitations. First, we conducted a retrospective analysis. While basic MRI sequences were consistent, acquisition parameters differed. Second, because of the fixed delays after contrast agent injection for the acquisition of post-contrast series, we may have missed optimal time windows for the characterization of some lesions, particularly in the arterial phase. Third, lesion-to-liver ratios were measured in ROIs and not volumetrically. Fourth, although readers were blinded, they were aware of the study design, which may have introduced detection bias. Fifth, the largest lesion of each subtype per patient was chosen for analysis, which could lead to bias.

In conclusion, Gd-EOB-enhanced MRI reproduces findings reported in the literature for ECA-enhanced MRI and may be a valuable tool for noninvasive HCC subtype differentiation according to the 5^th^ edition of the *WHO Classification of Digestive System Tumors*. This could help in identifying patients who may benefit from initial curative treatment or in selecting candidates for neoadjuvant strategies. Further understanding of the new HCC subtypes in the current WHO classification, and their implementation into diagnostic and therapeutic algorithms, may be a game changer for patients’ prognosis.

### Supplementary Information

Below is the link to the electronic supplementary material.

Supplementary file1 (PDF 147 KB)

## Abbreviations

AFP: Alpha-fetoprotein
APHE: Arterial phase hyperenhancement
chr-ST: Chromophobe subtype
cc-ST: Clear cell subtype
DWI: Diffusion-weighted imaging
ECA: Extracellular contrast agent
FS: Fat saturation
fib-ST: Fibrolamellar subtype
Gd-EOB: Gadoxetic acid
HBP: Hepatobiliary phase
HCA: Hepatocellular adenoma
HCC: Hepatocellular carcinoma
mt-ST: Macrotrabecular massive subtype
nos-ST: Not otherwise specified subtype
ROI: Region of interest
sc-ST: Scirrhous subtype
sh-ST: Steatohepatitic subtype

## Declarations

### Guarantor

The scientific guarantor of this publication is PD Dr Timo Auer.

### Statistics and biometry

No complex statistical methods were necessary for this paper.

### Methodology

• retrospective

• diagnostic or prognostic study

• performed at one institution
