# Supplementary material for: Gd-EOB MRI for HCC subtype differentiation in a western population according to the 5th edition of the World Health Organization classification
Source: Eur Radiol. 2023 Apr 28;33(10):6902–15. doi: 10.1007/s00330-023-09669-y (PMC10511376; doi:10.1007/s00330-023-09669-y)
Supplement: Supplementary file 1 — Supplementary file1 (PDF 147 KB) [file 330_2023_9669_MOESM1_ESM.pdf]

SUPPLEMENTARY MATERIALS

Sequence	Orientation	TR (ms)	TE (ms)	Flip Angle (°)	Matrix	Slice (mm)
T1 VIBE Dual Echo	Axial	6.6	2.4	10	320	3
T1 VIBE FS unenhanced	Axial	4.3	2.1	9	320	3
T1 VIBE FS (high flip angle) unenhanced	Axial	6.7	2.4	25	320	3
T1 VIBE FS arterial	Axial	4.3	2.1	9	320	3
T1 VIBE FS portovenous	Axial	4.3	2.1	9	320	3
T1 VIBE FS delayed	Axial	4.3	2.1	9	320	3
T2 HASTE	Axial	1400	94		352	6
T2 TSE FS	Axial	7964	102		512	6
DWI EPI (b = 50, 400, 800)	Axial	2000	55		128	6
T1 FL2D	Axial	195	4.8	70	320	6
T1 VIBE FS hepatobiliary	Axial	4.3	2.1	9	320	3
T1 VIBE FS high flip angle	Axial	6.7	2.4	25	320	3
T1 VIBE FS hepatobiliary	Coronal	7.1	2.4	30	288	2
T1 VIBE FS hepatobiliary	Sagittal	7.2	4.8	30	320	2

Supplementary Table: representative MRI acquisition parameters for Gd-EOB-enhanced MRI at 1.5T. All sequences except DWI are acquired in breath-hold. TR: repetition time. TE: echo time. VIBE: Volumetric interpolated breath-hold examination. FS: fat saturation. DWI: diffusion-weighted imaging. EPI: echoplanar imaging. HASTE: half fourier single-shot Turbo spin-Echo. FL2D: fast low angle shot, 2-dimensional.
